# Administration Methods of Mesenchymal Stem Cells in the Treatment of Burn Wounds

**DOI:** 10.3390/ebj3040043

**Published:** 2022-11-03

**Authors:** Astrid Bjørke Jenssen, Samih Mohamed-Ahmed, Esko Kankuri, Ragnvald Ljones Brekke, Anne Berit Guttormsen, Bjørn Tore Gjertsen, Kamal Mustafa, Stian Kreken Almeland

**Affiliations:** 1 Norwegian National Burn Center, Department of Plastic, Hand, and Reconstructive Surgery, Haukeland University Hospital, 5021 Bergen, Norway; 2 Center for Translational Oral Research (TOR), Tissue Engineering Group, Department of Clinical Dentistry, Faculty of Medicine, University of Bergen, 5020 Bergen, Norway; 3 Department of Pharmacology, Faculty of Medicine, University of Helsinki, 00014 Helsinki, Finland; 4 Department of Clinical Medicine, Faculty of Medicine, University of Bergen, 5020 Bergen, Norway; 5 Department of Anesthesia and Intensive Care, Haukeland University Hospital, 5021 Bergen, Norway; 6 Centre for Cancer Biomarkers CCBIO, Department of Clinical Science, University of Bergen, 5020 Bergen, Norway; 7 Department of Medicine, Hematology Section, Haukeland University Hospital, 5021 Bergen, Norway

**Keywords:** stem cells, mesenchymal stromal cells, mesenchymal stem cells, burns, wound healing, cell delivery, tissue engineering

## Abstract

Cellular therapies for burn wound healing, including the administration of mesenchymal stem or stromal cells (MSCs), have shown promising results. This review aims to provide an overview of the current administration methods in preclinical and clinical studies of bone-marrow-, adipose-tissue-, and umbilical-cord-derived MSCs for treating burn wounds. Relevant studies were identified through a literature search in PubMed and Embase and subjected to inclusion and exclusion criteria for eligibility. Additional relevant studies were identified through a manual search of reference lists. A total of sixty-nine studies were included in this review. Of the included studies, only five had clinical data from patients, one was a prospective case–control, three were case reports, and one was a case series. Administration methods used were local injection (41% in preclinical and 40% in clinical studies), cell-seeded scaffolds (35% and 20%), topical application (17% and 60%), and systemic injection (1% and 0%). There was great heterogeneity between the studies regarding experimental models, administration methods, and cell dosages. Local injection was the most common administration method in animal studies, while topical application was used in most clinical reports. The best delivery method of MSCs in burn wounds is yet to be identified. Although the potential of MSC treatment for burn wounds is promising, future research should focus on examining the effect and scalability of such therapy in clinical trials.

## 1. Introduction

Patients with major burns require demanding care, such as intensive care support, numerous surgical interventions, and rehabilitation over several years [1]. Such long-lasting disease burdens exert significant impact on patients and healthcare systems [2,3,4,5]. The main goal is wound healing, which is directly related to survival and functional outcomes [6]. Therefore, applying the best therapies to advance and facilitate wound healing is critical. Various cell-based therapies have been developed to expedite wound healing in chronic wounds and burns. Multiple cell types, mainly stem cells, fibroblasts, keratinocytes, and inflammatory cells, participate in the natural course of wound healing; hence, the effects of many different cell types have been evaluated [7,8,9]. Stem cell-based therapy of wounds is promising. Previous animal studies have found significantly improved healing of burn wounds treated with mesenchymal stem or stromal cells (MSCs) compared to controls [10]. MSCs have the potential to differentiate into various cell lineages, such as keratinocytes, adipocytes, chondrocytes, myocytes, and osteoblasts [11]. Their beneficial impact on wound healing can be attributed to different mechanisms; the cells can differentiate into functional skin components, produce factors and cytokines that stimulate nearby cells into tissue repair, and modulate immune responses to control inflammation [11]. There are indications that MSCs are involved in inducing regeneration of the skin’s histologic pattern, pigmentation, and appendages [12,13]. MSCs, especially adipose tissue-derived stem cells (ASCs), umbilical cord-derived stem cells (UC-MSCs), and bone marrow-derived stem cells (BM-MSCs), are easily obtained, elicit little to no immunogenic responses, and can be frozen with minimal loss of viability. These properties make them suitable for allogeneic as well as autologous therapeutic purposes [14]. Several administration methods, including local injection, topical cell suspension, topical cell scaffolding, and systemic injection or infusion, have been reported to deliver MSCs in treating burns and other wounds [10,15]. It seems reasonable that the administration method significantly impacts the wound-healing effects of MSCs. The properties and the composition of the microenvironment are major determining factors for both their differentiation and function [16].

The swift permanent coverage of large areas is critical in extensive burn wounds. The loss of integumental protection lowers body temperature, leads to the loss of fluids, and leaves the body susceptible to infections. The administration method of MSCs should provide effective delivery of the cells to the burn wound to aid wound closure. Moreover, in deep burns, the standard of care is surgical excision followed by the application of split-thickness skin grafts (STSGs). Thus, the delivery method of the MSCs should be compatible with STSG treatment and should not reduce the rate of graft-take or increase infection risk. These particular concerns in burn wound care can be crucial when searching for the preferred clinical method for MSC treatment of burn wounds.

We provide a literature review evaluating the currently preferred method for administering MSCs to burn wounds, including preclinical and clinical studies. We focus this review on ASCs, UC-MSCs, and BM-MSCs, as these are the most readily available mesenchymal stem cells for clinical use.

## 2. Methods

### 2.1. Data Sources and Searches

The literature search was performed (last updated on 30 September 2022) in PubMed and Embase and was restricted to English language papers up until September 2022. The search terms used were: (“Burn” OR “Burn injury” OR “Burn wounds” OR “Thermal injury” OR “Thermal burn”) AND (“Mesenchymal stem cells” OR “mesenchymal stromal cells” OR “Adipose stem cells” OR “Adipose tissue derived stromal cells” OR “Adipose tissue derived stem cells” OR “Bone marrow derived stem cells” OR “Bone marrow derived stromal cells” OR “Adipose derived stem cells” OR “Adipose derived stromal cells” OR “umbilical stem cells” OR “Wharton’s jelly” OR “Stem cells” OR “stromal cells” OR “MSC” OR “ASC” OR “BMMSC” OR “BMSC” OR “BM-MSC” OR “USC” OR “UCSC” OR “UC-MSC”) NOT (“Review”). Additional relevant articles were manually identified from the reference lists of included articles.

### 2.2. Study Selection

**Figure 1:**
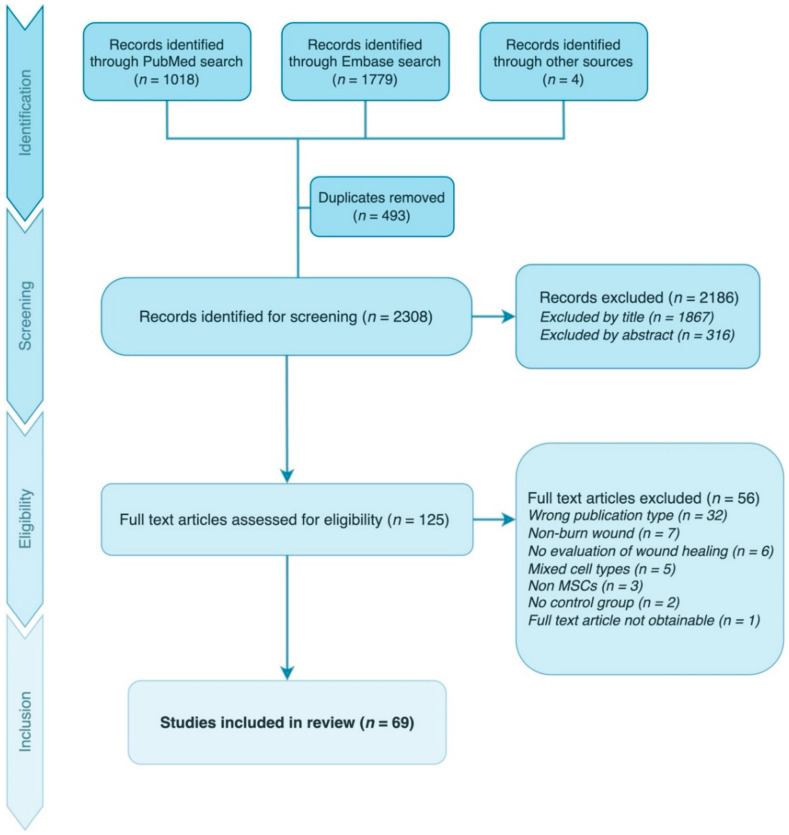
Illustration of the study selection process.

After identification, the studies were screened by title and abstract. Subsequently, the identified articles were reviewed in full text and determined for eligibility using the inclusion and exclusion criteria presented in Table 1. Unresolved issues were discussed by the first (A.B.J.) and last author (S.K.A.). The selection process is illustrated in Figure 1.

## 3. Results

### 3.1. Study Characteristics

**Figure 2:**
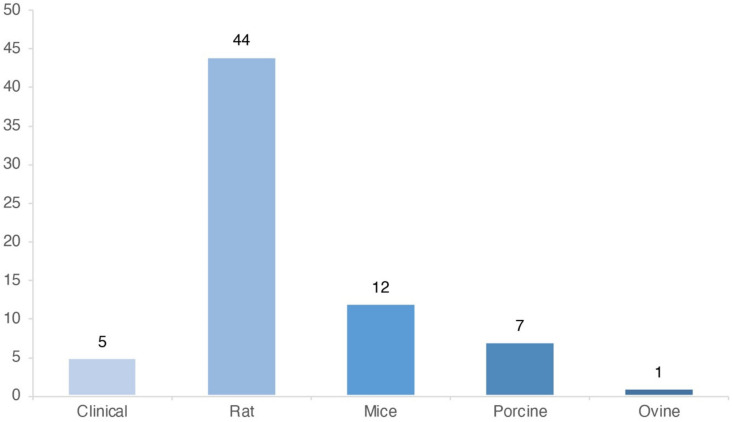
Study models used in included studies (*n* = 69).

Sixty-nine studies were included in the present review. The characteristics of the clinical studies included are listed in Table 2. The characteristics of the preclinical studies are presented in Table 3, Table 4 and Table 5. In the experimental model, rats were most frequently used (64%; 44/69), followed by mice (17%, 12/69) and porcine models (10%; 7/69) (Figure 2). There was considerable heterogeneity between administration methods, cell dosages, and the effect measures.

Only five clinical studies fulfilled the inclusion criteria. Of these, three were case reports, one a case series, and one a case–control study (Table 2). Two (40%, 2/5) of the included clinical studies combined MSC treatment with STSGs, as opposed to only 5% (3/64) of preclinical studies. Overall, the results most frequently reported were increased wound healing rate and earlier wound closure (in 78% of the studies, 54/69), faster re-epithelialization (in 38%, 26/69), and increased revascularization (in 45%, 31/69). No significant effect was found in 4% (3/69) of the studies, and adverse effects were reported only in 3% (2/69).

### 3.2. Clinical Studies

Three of the five clinical reports used topical application for cell delivery. One combined this with local injection, while the two remaining studies used either local injection or cell-seeded scaffolds (Table 2). One of the studies employed a fibrin sealant spray as the MSC administration method. The mean cell dose per cm^2^ when calculated from available data was 4.5 × 10^4^ (SD = 4.0 × 10^4^) (*n* = 3). All the studies used allogeneic MSCs. The treated wounds in these studies ranged from deep partial to full thickness burns. All clinical studies reported favorable outcomes. Rasulov et al. (2005) found an accelerated restitution of the patient after the topical application of MSCs and complete graft adherence to the wound after excision and grafting after MSC treatment [20]. Secondly, Mansilla et al. (2015) found the fibrin matrix spray delivery of allogenic BM-MSCs to increase epithelialization, graft-take of split-thickness skin grafts and wound closure of grafted areas in their clinical case [19]. Thirdly, a prospective comparative study by Abo-Elkheir et al., treating 60 patients randomized to (i) traditional treatment with excision and graft, (ii) treatment with excision and local injection of autologous BM-MSCs or (iii) allogenic UC-MSCs, found increased healing and reduced hospital length of stay [17]. Furthermore, this was the only study with clearly defined outcomes, such as rate of healing, complications, length of stay and treatment costs, and was the only one to report a control group with standard of care. They reported higher early complication rates in the UC-MSC group, but lower early complication rates in the BM-MSC group compared to the STSG group. Complication rates for late complications were lower in both MSC groups compared to the control (STSG group). The other four studies reported either a decreased level of complications or no complications but did not include a control group. No specific dose-related effects were reported in any of the studies.

### 3.3. Preclinical Studies

**Figure 3:**
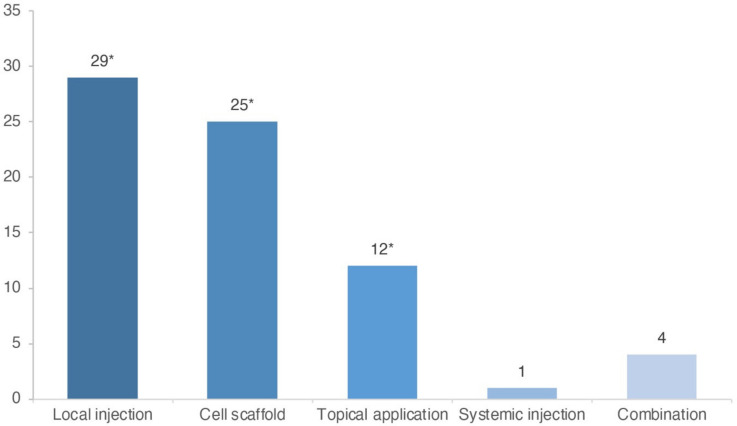
Administration methods in preclinical trials by category (*n* = 71). * Six studies compared different administration methods between study groups in the same experiment. Therefore, these six studies are represented as thirteen different groups in the figure representation.

Local injection (41%; 29/71) was the most common administration method in preclinical studies (Figure 3). For BM-MSCs and ASC, their use was mainly allogeneic and xenogeneic. However, three studies used autologous BM-MSCs, and one used autologous ASCs (Table 3 and Table 4). For UC-MSCs, all studies used xenogeneic cells (Table 5).

Four studies combined administration methods of MSCs in treating the same wound [46,47,69,70], while six studies compared administration methods in two or three separate study groups (Figure 3, Table 3, Table 4 and Table 5) [21,36,43,49,75,80]. Both intradermal and subcutaneous injections were commonly used. For injection, when specified, the most commonly used medium was phosphate-buffered saline (PBS, 38%; 11/29) (Table 3, Table 4 and Table 5). Only one study applied systemic injection of MSCs. Two of the three preclinical studies that used STSGs in combination with MSC therapy were a porcine model. Four of the porcine studies used topical application for cell delivery, one used cell-seeded scaffolds, and two used local injection (Table 3, Table 4 and Table 5). Due to heterogeneity between the studies, attaining consistent data on cell dosages was difficult. The mean cell dosage used was 4.6 × 10^5^ cells/cm^2^ (SD = 4.1 × 10^5^), when calculated as cells/cm^2^ from studies where these data were available (*n* = 27). Most studies utilized cells from passages 3–5 (Table 3, Table 4 and Table 5). There was substantial variation in the study models in terms of wound depth and size and animal species. Accordingly, the results were not standardized in terms of methodology and outcomes. Therefore, a direct comparison of dose-related effects could not be obtained.

## 4. Discussion

This review covered both clinical and preclinical use of MSCs in treating burn wounds. Most studies on this subject are preclinical. In general, the clinical studies found were limited and lacked methodological consistency. In line with previous reports in the field, local injection was the most frequently reported administration method overall [10]. However, topical application was the most frequently used method in clinical studies.

There are some distinct requirements when considering using MSCs for treating major burns, as opposed to treating minor traumatic wounds or chronic and diabetic wounds. Mainly, there is a larger surface area to cover, and the time in each surgical procedure should be reduced to a minimum to limit fluid and blood loss and the risk of peri-operative hypothermia. The benefit of local injection of MSCs is the precise delivery of the cells directly into the wound, where needed, in a specified dosage. In clinical practice, however, injection techniques may vary between operators, and procedures could become time-consuming when treating larger areas. The time spent in burn surgery has been shown to correlate inversely with patient outcomes [84]. Consequently, spray delivery may be particularly interesting as it allows for the easy scalability of the administration method to any wound size. Cell-based therapies with autologous skin cell suspensions have been successfully used to treat more extensive burn wounds, commercially available as a skin spray [8]. Skin spray delivery systems have also been reported with a fibrin sealant in combination with cells [85,86,87,88]. Fibrin sealant formulations are regularly used in surgical settings worldwide [85,89]. When combined with MSCs, fibrin sealants seem compatible with cell viability and proliferation, and fibrin–MSCs combinations have successfully been applied to wounds through the spray method [87]. However, the fibrinogen/thrombin ratio is crucial to enable an optimal 3D clot microstructure allowing for the proliferation of MSCs [90]. Additionally, the cells delivered with fibrin sprays must be considered non-protected from the wound environment, and the cell dosage is more difficult to standardize and monitor [91]. In contrast, cell scaffolds allow for a predictable cell dosage and optimization of the microenvironment for cell proliferation. A comprehensive review of stem cell treatment for various wounds found cell scaffolding to be the preferred method of administration due to the possibility of optimizing the 3D microenvironment, in which additional components, such as growth factors, can be added to the scaffolds [7]. The cell scaffolds can protect the cells from the harsh environment of the wound and preserve the “stem-ness” of the cells [91]. A recent review by Mamsen et al. found that the viability of ASCs improved with an application through ASC-imbedded scaffolds, increasing neovascularization compared to ASC injection [92]. This is in line with findings from the preclinical study by Barrera et al., which included both injection and hydrogel scaffold administration of ASCs and found that the scaffold facilitated better burn wound healing compared to injection [49]. In addition to this study, we found five preclinical studies comparing administration methods in their experiments [21,36,43,75,80]. Overall, the prevalent findings had better results when using cell-seeded scaffolds compared to either topical administration or local injection.

Systemic intravenous injection, or infusion, of MSCs, was used in only one of the included studies in this review. Although MSCs have the ability to migrate and home to the damaged tissue, their homing to the targeted site is not inevitable. They have been shown to accumulate in various tissues after injection, especially the lungs [93]. The safety of this approach for clinical use is not established. For instance, MSCs have been described to have pro-coagulant properties, and thromboembolic events have been reported in conjunction with systemic MSC treatment in patients [14,94,95]. Additionally, MSCs may affect the immune system and systemic inflammation [14]. Whether these effects are indeed beneficial or detrimental is uncertain, though a recent meta-analysis found MSC treatment to be safe with very few risks involved [96].

The clinical studies included in this review mainly consisted of case reports and were not found sufficient to determine the clinical effects of MSC therapy. In one study including 60 patients, MSC therapy was combined with surgical excision and compared to the standard of care, which is excision and autologous STSGs [17]. The authors report comparable results and fewer complications with MSC therapy, without STSG, compared to the group treated with STSGs without MSC therapy, though with a high complication rate in the excision + STSG group. Especially interesting in this study was the finding of higher complication rates in the group using UC-MSCs compared to BM-MSCs. Seventy percent of patients suffered early complications in the form of infection in the UC-MSC group, compared to 25% in the BM-MSC group. Although a very interesting finding, the authors do not provide a rationale for the high infection rate in the UC-MSC group compared to the BM-MSC group. However, the early complication rate was also high in the control group (excision + STSG), with 50% of patients experiencing either infection, partial loss of graft, or both [17]. These findings indicate a somewhat high complication rate overall that might limit the interpretation and generalizability of this study.

Combining MSCs and STSGs could become relevant in a clinical setting, either as part of a bioengineered product or to facilitate the healing potential in the direct treatment of the area to be covered with STSGs. More importantly, when considering the administration method of MSCs in treating extensive burns, combining MSCs with STSGs in one way or another seems a natural first step. Since STSGs depend on neo-vascularization from the wound bed, MSCs would need to be delivered without compromising the neo-vascularization of the skin graft. ASC-imbedded scaffolds combined with STSGs, specifically PEG-fibrin hydrogel over meshed STSG, have been reported to increase vascularization and do not seem to impair graft take [52]. However, only a few studies have examined the combined effects of MSC treatment and STSGs. In fact, only two of the five clinical reports combined topical MSC treatment with autologous skin grafts. Furthermore, in both studies, there was an interval of 4–35 days from the MSC application to skin grafting. Mansilla et al. combined a fibrin sealant spray MSC treatment with meshed skin grafts 35 days after MSC application due to a lack of complete wound healing by MSCs alone [19]. Even though the skin grafts were applied with some delay, the authors did report improved re-epithelialization between the skin bridges of the STSGs, and the healed skin had an appearance closer to normal skin compared to their previous experiences with meshed grafts. This finding led them to hypothesize that the future use of MSCs could be combined with wide-meshed STSGs. However, there was no standardized control group comparison to support the findings. Rasulov et al. also applied skin grafts after topical MSC treatment. They compared two areas, STSG-transplanting either 4 or 13 days after MSC treatment, and reported 99% and 100% graft-take after ten days post-op, respectively [20]. The discrepancy between the experimental model of clinical studies versus preclinical studies is interesting. Although the numbers are small, this might imply that the gap from preclinical to clinical trials is yet to be bridged. Novel treatment methods have not yet been implemented in replacement for standard treatment in clinical trials. Further clinical implementation seems to rely on a combination of standards of care and novel therapies as the first step in increasing the potential of novel cell therapies.

Murine models were most frequently used as an experimental model in preclinical studies. Only seven studies used a porcine model (Figure 2). It is well known that experimental mouse models poorly resemble human inflammatory processes and wound healing [97,98]. The fact that mouse models represented 19% (12/64) of the included preclinical experiments underlines the need to move towards more clinically relevant models. Porcine skin resembles human skin better in both composition and the process of wound healing [98]. As a result, porcine studies would probably be more clinically relevant and could represent an apt animal model for further research focus.

There is some uncertainty regarding the ideal cell dosage for promoting wound healing. Higher doses (>20% of MSCs relative to fibroblasts) of MSCs seem to inhibit fibroblast migration, whereas lower doses (10% of MSCs relative to fibroblasts) enhance it in in vitro studies [99]. We found that there is little consistency in the cell dosages used in the current literature. Moreover, the cell dosages also vary between the methods of administration. Burmeister et al. found an increased diameter of blood vessels regenerated in the wound related to cell dosage. Notably, they also demonstrated a dose-dependent increase in collagen deposition [52]. Whether the increased collagen deposition is beneficial for healing or problematic for scarring is yet to be fully understood. The mechanisms through which these changes occur are not well described and will undoubtedly be important in the future development of new treatment techniques.

An aspect of MSC treatment is the possible use of MSCs with already established commercial wound healing products. Pairing MSC treatment with current treatment methods would make MSC treatment easier to implement and more accessible for widespread clinical use. A study not included in this review, due to its use of embryonic stem cells (ESC) as opposed to BM-MSCs, UC-MSCs, or ASCs, indicates that ESCs adhered well to Integra™ and were effectively delivered to the burn wound bed when using Integra™ as the cell scaffold [100]. Such combined use of novel methods and commercially available products is an exciting possibility for future developments that might speed up the transition from bench to bedside.

Comparing ASCs to BM-MSCs, ASCs are more readily available and easy to harvest for allogeneic use through liposuction [101]. They have demonstrated similar capacities for improved healing in the zone of stasis as BM-MSCs [102]. Allogeneic MSCs are considered easier to use in a clinical setting, as they can be readily available as an off-the-shelf product and do not require the expansion time needed for autologous MSCs. This is important, especially when treatment is administered to prevent initial burn progression in the acute phase—none of the clinical reports identified in this review utilized autologous cells (Table 2). Nevertheless, there can be wound healing advantages in using autologous versus allogeneic stem cells. In preclinical settings, Shumakov et al. reported significantly increased wound healing in the group receiving autologous BM-MSCs compared to allogeneic BM-MSCs. However, both groups had increased wound healing compared to the baseline control group [44]. It remains to be investigated whether a similar response could also be obtained for the ASC treatment. For autologous use, bone marrow might be as readily harvested as adipose tissue, considering the risk that adipose tissue may be compromised in burn patients. Additionally, BM-MSCs can be isolated from bone marrow aspirated from the iliac crest by a procedure not considered very invasive, though with some risk of infection. UC-MSCs are also readily harvested from disposed umbilical cords and require no invasive procedures, making them an attractive source of allogeneic MSCs. Animal models showed comparable improvements in wound healing to other MSC sources [75,76,77,78,79,80,81,82,83]. However, in the clinical study by Abo-Elkheir et al., the UC-MSC group was related to higher complication rates [17]. Whether this is related to their overall high complication rate in their study, or a true finding inherently related to specific features of UC-MSCs, is yet to be investigated.

A promising implementation of stem cell treatment is through the novel field of skin bioprinting. This consists of developing tissue-engineered skin substitutes to deliver beneficial growth factors and cells via a matrix to promote wound healing. The matrix serves as a scaffold for tissue regeneration and promotes the formation of new autologous skin. Three-dimensional printing of biological materials makes it possible to layer several different types of matrices and cells, mimicking natural skin layers. One might argue that fibrin sealant spray systems are, in fact, a method of in situ 3D bioprinting when combined with the delivery of stem cells and keratinocytes, depending on the dose, thickness and matrix density. The fibrin matrix can also be conjugated with additional factors, such as growth factors, to stimulate tissue regeneration [103,104].

There is an apparent lack of clinical studies on MSCs for burn wound healing. Clearly, there is a need for further clinical testing and validation. The few clinical studies conducted thus far have shown that MSCs can safely be administered to the patient. Positive effects have been reported. However, the generalizability of the findings in the current literature is limited due to the predominance of case reports and preclinical studies. Randomized controlled trials are needed to determine the clinical effects of MSC treatment for burn wounds.

## 5. Conclusions

In preclinical studies, MSCs are most commonly administered to burn wounds through local injection, either intradermally or subcutaneously. In clinical trials and cases, topical application remains the most common administration method. Future research should focus on the preferred and scalable administration method, optimal cell dosages and combined therapies to facilitate the translation of MSC therapy into clinical trials and practice in burn wound treatment.

## Figures and Tables

**Table 1:** Inclusion and exclusion criteria.

Inclusion Criteria	Exclusion Criteria
English language Original scientific studies In vivo studies Bone-marrow-, umbilical-cord-, or adipose-tissue-derived stem cells Cutaneous burn wounds	Not in English Review articles In vitro studies Radiation or chemical burn studies Other types of MSCs Non-MSC cells included in the treatment group Use of further differentiated MSCs Genetic alteration of MSCs beyond genetic marking

**Table 2:** Characteristics of studies included—clinical studies (*n* = 5).

Authors	Study Model	Patient Characteristics (*n*, Age, Sex, TBSA)	Burn Depth	Cell Species	Groups	Cell Delivery, Medium	Cell Dosage		Results (End Points)
							Dose (Passage)	Cells/ cm^2 1^	
Abo-Elkheir et al. (2017) [17]	Prospective case–control	*n* = 60, 18–35 y, male and female, 10–25% TBSA IC: Both sexes, age 15–50 y, TBSA 10–25% EC: Comorbidity, superficial or old burns, chemical, radiation or electric burns	Full thickness	Al BM-MSC, Al UC-MSC	Excision and STSG, excision and BM-MSC, excision and UC-MSC	Local injection, n/a	1 × 10^5^ cells/cm^2^ (n/a)	1 × 10^5^	Increased rate of wound healing compared to STSG group, in both MSC groups, and shorter length of stay. Less early complications in BM-MSC group; infection was seen in 25% of the patients, but higher early complication rates in UC-MSC group; infection in 70%. Early complication rate was 50% in excision + STSG group. A total of 95% of patients in STSG group had late complications, compared to 45% in BM-MSC group and 30% in UC-MSC group. (Rate of burn healing, early and late complications, hospital stay length, costs)
Jeschke et al. (2019) [18]	Case report	*n* = 1, male, mid-twenties, >70% TBSA 18 months after injury	Full thickness	Al UC-MSC and commercial Al Ch-MSC		Topical application and injection, fibrin sealant and Ringer’s lactate	3 × 10^6^ cells/mL in topical solution (n/a)	n/a	Rapid re-epithelialization. Reduction in wound percentage and healing of infections. Limited scarring over 6 years and no adverse effects. (Effect on burn wounds with delayed healing)
Mansilla et al. (2015) [19]	Case report	*n* = 1, 26 y, male 60% TBSA, 30% full thickness	Full thickness	Al cadaveric BM-MSC	n/a	Topical application, fibrinogen and thrombin spray	1 × 10^4^ cells/cm^2^ (P2)	1 × 10^4^	Rapid epithelialization, more normal skin appearance compared to previous experiences in the burn unit. No adverse effects. (Safety)
Rasulov et al. (2005) [20]	Case report	*n* = 1, 45 y, female 40% TBSA, 30% full thickness	Deep partial and full thickness	Al BM-MSC	n/a	Topical application, n/a	2–3 × 10^4^ cells/cm^2^ (n/a)	2.5 × 10^4^	Rapid epithelialization. Increased angiogenesis and granulation. Pain relief. Good graft-take of STSGs. (Neo-angiogenesis and graft take)
Wittig et al. (2020) [13]	Case series	*n* = 5, 2–58 y, male TBSA 12–55% IC: Age >= 2 y, full thickness burns, not healed within >= 21 days EC: Infection	Deep partial and full thickness	Al BM-MSC	n/a	Cell scaffold, pre-clotted PRP and thrombin	1–3 × 10^7^ cells per patient (n/a)	n/a	Early granulation tissue, rapid re-epithelialization. Full healing in 1–5 months. Recovery of pigmentation. Slight discoloration of healed skin, less hypertrophy, and contractures. (Effect on burn wounds with delayed healing)

Abbreviations: TBSA = total body surface area, y = years, IC = inclusion criteria, EC = exclusion criteria, BM-MSC = bone-marrow-derived stem cell, UC-MSC = umbilical-cord-blood-derived stem cells, Ch-MSC = Chorion-derived MSCs, Al = allogeneic, STSG = split thickness skin graft, n/a = no available data, PRP = platelet-rich plasma. ^1^ Cell dosage recalculated as cells/cm^2^ of either wound area or scaffold when size was stated.

**Table 3:** Characteristics of studies included—preclinical studies using bone-marrow-derived MSCs (*n* = 27).

Authors	Animal Model (*n*)	Burn Depth	Cell Species	Administration Method, Medium	Cell Dosage		Results in MSC Group
					Dose (Passage)	Cells/cm^2 1^	
A V et al. (2020) [21]	Rat (n/a)	Partial thickness	Xe BM-MSC, human	Cell scaffold and topical application (2 groups), hydrogel and DMEM	1 × 10^6^ cells (P3–5)	n/a	Increased wound contraction. Earlier wound closure, but only in scaffold group. No effect in topical MSC group.
Abdel-Gawad et al. (2021) [22]	Rat (90)	Partial thickness	Al BM-MSC	Subcutaneous injection, DMEM	2 × 10^6^ cells/mL (n/a)	n/a	Increased wound healing. Reduced scar formation.
Alapure et al. (2018) [23]	Mice (n/a)	Full thickness	Al BM-MSC	Cell scaffold, ACgel scaffold	1 × 10^5^ cells/scaffold (P3–5)	5.1 × 10^5^	Increased wound closure rate, re-epithelialization, blood vessel growth and granulation.
Caliari-Oliveira et al. (2016) [24]	Rat (134)	Full thickness	Xe BM-MSC, Mice	Intradermal injection, PBS	5 × 10^6^ cells/wound (P3–4)	1.1 × 10^5^	Increased epithelialization after 60 days.
Clover et al. (2015) [25]	Porcine (3)	Deep partial thickness	Al BM-MSC	Topical application, fibrin sealant (Tisseel™)	4.5 × 10^6^ cells/wound (P4)	1 × 10^6^	Increased wound healing. Increased collagen density, increased epidermal area and dermal thickness.
Fu et al. (2006) [26]	Porcine (6)	Deep partial thickness	Au BM-MSC	Local injection, n/a	2 × 10^6^ cells/wound (n/a)	n/a	Faster re-epithelialization, increased vascularization and collagen.
Guo et al. (2016) [27]	Rat (49)	Deep partial thickness	Al BM-MSC	Cell scaffold, small intestinal submucosa	5 × 10^5^ cells/cm^2^ (P3)	5 × 10^5^	Accelerated wound closure and granulation, vascularization and neo-epidermal cells.
Ha et al. (2010) [28]	Rat (32)	Partial thickness	Al BM-MSC	Intradermal injection, saline solution	n/a (n/a)	n/a	Earlier wound closure.
Hosni Ahmed et al. (2017) [29]	Rat (72)	n/a	Al BM-MSC	Local injection, PBS	1 × 10^6^ cells/mL (P3)	n/a	Accelerated wound healing.
Imam et al. (2019) [30]	Rat (40)	Full thickness	Al BM-MSC	Local injection, PBS	1 × 10^6^ cells/cm^2^ (P3)	1 × 10^6^	Increased wound healing and epithelialization.
Imbarak et al. (2021) [31]	Rat (60)	Deep partial thickness	Al BM-MSC	Intradermal injection, PBS	1 × 10^6^ cells/wound (n/a)	n/a	Accelerated wound healing, increased epidermal thickness. Regenerated hair follicles.
Liu et al. (2008) [32]	Porcine (24)	Deep partial thickness	Au BM-MSC	Cell scaffold, collagen-GAG	2 × 10^6^ cells/mL (P2–5)	n/a	Better healing and keratinization, less wound contraction. Increased vascularization. No adverse effects.
Lykov et al. (2017) [33]	Rat (25)	Partial thickness	Al BM-MSC	Local injection, n/a	2 × 10^5^ cells/wound (P2–4)	n/a	Decrease in defect skin area, increased re-epithelialization and wound closure rate.
Mansilla et al. (2010) [12]	Porcine (1)	Full thickness	Xe BM-MSC, rabbit	Topical application, fibrin sealant	2 × 10^6^ cells/mL/cm^2^ (n/a)	n/a	Increased granulation, vascularization, healing of wound with skin appendages.
Mohajer Ansari et al. (2020) [34]	Rat (48)	Deep partial thickness	Al BM-MSC	Intradermal injection, PBS	1 × 10^6^ cells (n/a)	4.4 × 10^5^	Increased biomechanical strength of wound, increased wound closure rate, increased epithelialization, increased remodeled collagen content, increased angiogenesis.
Oh et al. (2018) [35]	Mice (30)	Full thickness	Al BM-MSC	Systemic injection, n/a	5 × 10^5^ cells/mouse (n/a)	n/a	MSC migration to burn wound and increased wound healing.
Palakkara et al. (2020) [36]	Rat (105)	Full thickness	Al BM-MSC	Cell scaffold and local injection, Chitosan powder and decellularized porcine SIS (two groups)	1 × 10^6^ cells/wound (P3)	n/a	Increased angiogenesis and re-epithelialization. Best results in scaffold group.
Paramasivam et al. (2021) [37]	Rat (75)	Full thickness	Al BM-MSC	Cell scaffold, acellular porcine bladder	2.5 × 10^6^ cells/scaffold (P3)	n/a	Increased rate of healing. Increased granulation and early angiogenesis. Increased and more regular collagen deposition.
Rasulov et al. (2006) [38]	Rat (30)	Deep partial thickness	Al BM-MSC	Topical application, n/a	2 × 10^4^ cells/wound (n/a)	n/a	Increased angiogenesis and granulation.
Revilla et al. (2016) [39]	Rat (12)	Full thickness	Al BM-MSC	Local injection, n/a	2 × 10^6^ cells/wound (n/a)	n/a	Faster wound healing, increased collagen type 1. No infection in MSC group.
Revilla et al. (2018) [40]	Rat (10)	Full thickness	Al BM-MSC	Local injection, n/a	2 × 10^6^ cells/wound (n/a)	8.9 × 10^5^	Accelerated wound closure, good healing quality.
Revilla et al. (2020) [41]	Rat (30)	Full thickness	Xe BM-MSC, human	Subcutaneous injection, n/a	2 × 10^6^ cells/mL (n/a)	n/a	Accelerated wound healing, increased re-epithelialization.
Rodriguez-Menocal et al. (2022) [42]	Porcine (4)	Full thickness	Al BM-MSC	Local injection, n/a	n/a (P1)	n/a	Reduced wound contraction, less collagen type I/III deposition. Reduced scarring.
Sharifi et al. (2021) [43]	Rat (48)	Partial thickness	Al BM-MSC	Cell scaffold and local injection (3 groups), Aloe vera gel, chitosan-based gel and n/a	2 × 10^6^ cells/wound (n/a)	n/a	Earlier wound closure. Increased angiogenesis and granulation.
Shumakov et al. (2003) [44]	Rat (40)	Full thickness	Au and Al BM-MSC	Topical application, n/a	2 × 10^6^ cells/wound (n/a)	n/a	Increased wound closure rate, most in Au group. Increased angiogenesis and granulation.
Wu et al. (2021) [45]	Rat (n/a)	Deep partial thickness	Al BM-MSC	Intradermal injection, n/a	1 × 10^6^ cells/wound (P5–7)	n/a	Increased wound closure rate and healing.
Xue et al. (2013) [46]	Mice (60)	Full thickness	Xe BM-MSC, human	Intradermal injection and topical application, PBS and growth factor reduced matrigel	1 × 10^6^ cells/wound (n/a)	n/a	Increased wound healing and angiogenesis. Faster wound closure. Found MSCs in other tissues than treated.

Abbreviations: BM-MSC = bone-marrow-derived stem cell, Al = allogeneic, Au = autologous, Xe = xenogeneic, n/a = no available data, PBS = phosphate-buffered saline, DMEM = Dulbecco’s modified Eagle’s medium, SIS = Small intestinal submucosa. ^1^ Cell dosage recalculated as cells/cm^2^ of either wound area or as scaffold when size was stated.

**Table 4:** Characteristics of studies included—preclinical studies using adipose-tissue-derived MSCs (*n* = 28).

Authors	Animal Model (*n*)	Burn Depth	Cell Species	Administration Method, Medium	Cell Dosage		Results in MSC Group
					Dose (Passage)	Cells/cm^2 1^	
Alemzadeh et al. (2020) [47]	Rat (12)	Full thickness	Al ASC	Topical application and local injection around wound, hyaluronic acid hydrogel, covered with ADM	1 × 10^6^ cells/wound (P3–5)	1.3 × 10^6^	Increased wound closure rate. Reduced inflammation, increased angiogenesis and granulation.
Andrade et al. (2020) [48]	Rat (96)	Full thickness	Xe ASC	Intradermal injection, n/a	1.5 × 10^6^ cells/wound (P4–5)	2.1 × 10^5^	Increased wound closure rate.
Barrera et al. (2021) [49]	Mice (32)	Partial thickness	Al ASC	Cell scaffold and injection (2 groups), collagen–pullulan hydrogel and n/a	2.5 × 10^5^ cells/wound (P0–2)	n/a	Accelerated wound healing in scaffold group. Increased vascularization.
Bliley et al. (2016) [50]	Mice (24)	Full thickness	Xe ASC, human	Subcutaneous injection, PBS	6.8 × 10^6^ cells/wound (P3)	n/a	No statistical difference in wound closure times. ASC enhanced vascularization, collagen deposition and adipocyte differentiation. Increased hair follicle regeneration.
Boukani et al. (2022) [51]	Rat (36)	Full thickness	Al ASC	Cell scaffold, decellularized dOSIS	n/a (P3)	n/a	Increased wound closure rate, increased angiogenesis and collagen deposition. Multi-layer epidermis in MSC group.
Burmeister et al. (2018) [52]	Porcine (6)	Deep partial thickness	Al ASC	Topical application, FPEG hydrogel (fibrin-based)	1 × 10^5^, 5 × 10^5^ and 1 × 10^6^ cells/wound, 3 groups (n/a)	7.6 × 10^4^	Increased size of blood vessels and collagen deposition dose-related to ASC.
Cabello-Arista et al. (2022) [53]	Mice (25)	Full thickness	Xe ASC, human	Cell scaffold, radiosterilized human amnion and pig skin	6 × 10^4^ cells/cm^2^ (n/a)	6 × 10^4^	No effect on wound closure. Increased collagen deposition.
Chen et al. (2017) [54]	Rat (15)	n/a	Al ASC	Subcutaneous injection, PBS	1 × 10^6^ cells/wound (n/a)	1.4 × 10^5^	Accelerated wound healing rate.
Chung et al. (2016) [55]	Rat (n/a)	Full thickness	Al ASC	Cell scaffold, PEGylated fibrin gel	4 × 10^5^ cells/gel (P3–5)	7.6 × 10^4^	Earlier neovascularization. Better tissue organization.
Costa de Oliveira Souza et al. (2021) [56]	Rat (70)	Deep partial thickness	Al ASC	Cell scaffold, nanostructured cellulose–gellan–xyloglucan–lysozyme dressing	1 × 10^3^ cells/cm^2^ (n/a)	1 × 10^3^	Increased wound healing
Dong et al. (2020) [57]	Mice (15)	Deep partial thickness	Al ASC	Topical application, conformable hydrogel	3 × 10^5^ cells/wound (P3–5)	n/a	Significantly increased healing rate and accelerated wound closure. Enhanced neovascularization, reduction in scar formation.
Feng et al. (2019) [58]	Rat (12)	Deep partial thickness	Al ASC	Intradermal injection, PBS	5 × 10^5^ cells/wound (P3)	5 × 10^5^	Increased healing at all time points, vascular density and percentage of live follicles.
Franck et al. (2019) [59]	Rat (23)	Full thickness	Al ASC	Intradermal injection, n/a	3.2 × 10^6^ cells/wound (n/a)	6.6 × 10^5^	Increased wound healing and collagen deposition. Decreased lymphatic vessels. No significant difference in vascular amt.
Fujiwara et al. (2020) [60]	Ovine (7)	Full thickness	Al ASC	Topical application, PBS	7 × 10^6^ cells/wound (P4)	2.8 × 10^5^	Improved graft-take and graft size. Increased blood flow and epithelialization.
Gholipourmalekabadi et al. (2018) [61]	Mice (75)	Full thickness	Al ASC	Cell scaffold, decellularized human amniotic membrane	1 × 10^4^ cells/scaffold (P2)	1.3 × 10^4^	Accelerated wound healing, reduced scarring, increased neo-vascularization and re-epithelialization.
Karimi et al. (2014) [62]	Mice (40)	Full thickness	Al ASC	Local injection, n/a	1 × 10^6^ cells/mL (n/a)	n/a	Not statistically significant improvements.
Karina et al. (2019) [63]	Rat (28)	Partial thickness	Xe ASC, human	Intradermal injection, saline	4 × 10^5^ cells (P1)	n/a	Increased wound closure rate, but delayed wound closure at end of study compared to the control. Increased re-epithelialization, larger and more prominent skin appendages, increased angiogenesis.
Karina et al. (2021) [64]	Rat (30)	Deep partial thickness	Xe ASC, human	Intradermal injection, n/a	4 × 10^5^ cells/rat (P1)	n/a	Increased wound healing rate. Increased differentiation of healed skin. Increased vascularization. Not accelerated epithelialization.
Loder et al. (2014) [65]	Mice (20)	Partial thickness	Al ASC	Subcutaneous injection, PBS	1 × 10^6^ cells/wound (P3+)	n/a	Decreased wound depth, decreased apoptosis, increase in vascularization (not significant).
Lu et al. (2020) [66]	Rat (25)	Partial thickness	Xe ASC, human	Topical application, gelatin hydrogel and suspension	n/a (n/a)	n/a	Increased wound closure rate, most in group using hydrogel compared to cell suspension. Increased epidermal thickness.
Motamed et al. (2017) [67]	Rat (32)	Full thickness	Xe ASC, human	Cell scaffold, human amniotic membrane	5 × 10^5^ cells/cm^2^ (P3)	5 × 10^5^	Increased wound closure rate, lower inflammatory cell infiltration. Most healing in the first 14 days.
Ng et al. (2021) [68]	Mice (42)	Full thickness	ASC, n/a	Topical application, gellan gum-collagen hydrogel	6 × 10^4^ cells/wound (P3–5)	n/a	Increased wound healing and closure rate.
Oryan et al. (2019) [69]	Rat (48)	Full thickness	Al ASC	Intradermal injection and topical application, Aloe vera hydrogel	1 × 10^6^ cells/wound (P3–5)	1.3 × 10^6^	Increased rate of healing, less inflammation.
Oryan et al. (2019) [70]	Rat (48)	Full thickness	Al ASC	Intradermal injection and topical application, honey	1 × 10^6^ cells/wound(P3–5)	n/a	Increased angiogenesis, re-epithelialization and granulation.
Roshangar et al. (2021) [71]	Rat (36)	Full thickness	Al ASC	Cell scaffold, 3D-printed collagen and alginate scaffold	n/a (n/a)	n/a	Accelerated wound contraction and healing. Increased re-epithelialization, and multi-layer epidermis.
Shokrgozar et al. (2012) [72]	Rat (10)	Full thickness	Al ASC	Cell scaffold, collagen–chitosan	n/a (n/a)	n/a	Increased wound healing rate, increased epithelialization.
Wu et al. (2021) [73]	Mice (32)	Full thickness	Al ASC	Cell scaffold, 3D GS alginate hydrogel	2 × 10^6^ cells/scaffold (P3–5)	8.9 × 10^5^	Faster epithelialization. Increased angiogenesis and collagen deposition.
Zhou et al. (2019) [74]	Rat (27)	Full thickness	Au ASC	Subcutaneous injection, n/a	2 × 10^6^ cells/wound P3)	1 × 10^6^	Increased wound healing and angiogenesis.

Abbreviations: ASC = adipose-derived stem cell, Al = allogeneic, Au = autologous, Xe = xenogeneic, n/a = no available data, PBS = phosphate-buffered saline, ADM = acellular dermal matrix, dOSIS = ovine small intestinal submucosa. ^1^ Cell dosage recalculated as cells/cm^2^ of either wound area or as scaffold when size was stated.

**Table 5:** Characteristics of studies included—preclinical studies using Wharton’s-Jelly- or umbilical-cord-derived MSCs (*n* = 9).

Authors	Animal Model (*n*)	Burn Depth	Cell Species	Administration Method, Medium	Cell Dosage		Results in MSC Group
					Dose (Passage)	Cells/cm^2 1^	
Afzali et al. (2022) [75]	Rat (40)	Superficial partial thickness	Xe UC-MSC, human	Cell scaffold and local injection, PRP cryogel and cell culture medium (two groups)	2 × 10^6^ cells (n/a)	n/a	Improved wound healing, increased wound closure rate, best results in scaffold group. Increased re-epithelialization and increased early neo-angiogenesis.
Cheng et al. (2020) [76]	Porcine (4)	Full thickness	Xe WJ-MSC, human	Topical application, in situ fibrin–HA bioink	1 × 10^6^ cells/mL (P1)	n/a	Better healing with less inflammation, scarring and contraction. Increased re-epithelialization, better archeology. No infection.
Gholipour-Kanani et al. (2012) [77]	Rat (12)	Full thickness	Xe WJ-MSC, human	Cell scaffold, Cs:PVA nanofibrous web	4 × 10^4^ cells/scaffold (P1)	1.8 × 104	Accelerated wound healing and wound closure rate. Less inflammation. Increased re-epithelialization and granulation, regular pattern of regenerated collagen.
Gholipour-Kanani et al. (2014) [78]	Rat (12)	Full thickness	Xe WJ-MSC, human	Cell scaffold, PCL:Cs:PVA nanofibrous web	4 × 10^4^ cells/scaffold (P1)	4.2 × 10^4^	Accelerated healing process, but longer than non-burn wound group. Increased collagen deposition, granulation, and re-epithelialization. No complications reported.
Hashemi et al. (2020) [79]	Rat (32)	Full thickness	Xe WJ-MSC, human	Cell scaffold, HAM	1 × 10^6^ cells/scaffold (P3)	n/a	Increased rate of healing, re-epithelialization, granulation. Mature and organized scar tissue, less hemorrhage and inflammation.
Jehangir et al. (2022) [80]	Rat (35)	Partial thickness	Xe WJ-MSC, human	Cell scaffold, A-PCL composite scaffold and collagen (two groups)	1 × 10^5^ cells/cm (P1)	1 × 10^5^	Increased wound healing and complete epithelialization in both MSC groups, best in A-PCL-WJ-MSC group with complete epidermal restoration and near normal skin appendage regeneration. Wound infection in one animal in the collagen-WJ-MSC group.
Nazempour et al. (2020) [81]	Rat (40)	Full thickness	Xe WJ-MSC, human	Cell scaffold, ADM	2 × 10^6^ cells/scaffold (n/a)	n/a	Increased wound closure rate, angiogenesis, granulation, and epithelialization.
Pourfath et al. (2018) [82]	Rat (24)	Full thickness	Xe WJ-MSC, human	Topical application, cell spray + sterile gauze Vaseline covering	5 × 10^5^ cells/wound (P3)	n/a	Increased re-epithelialization and granulation, decreased hemorrhage and inflammation.
Zhang et al. (2015) [83]	Rat (84)	Full thickness	Xe WJ-MSC, human	Subcutaneous injection, saline	2 × 10^6^ cells/rat (P2–4)	n/a	Significantly higher wound healing rate, shorter wound healing time. Lower increase in inflammatory cytokines.

Abbreviations: WJ-MSC = Wharton’s-Jelly-derived mesenchymal stem cell, UC-MSC = umbilical-cord-derived stem cell, Xe = xenogeneic, n/a = no available data, ADM = acellular dermal matrix, HA = hyaluronic acid, Cs:PVA = Chitosan-poly(vinyl alcohol), PCL:Cs:PVA = Poly(caprolactone)-chitosan-poly(vinyl alcohol, HAM = Human amniotic membrane, A-PCL = aloe vera-polycaprolactone. ^1^ Cell dosage recalculated as cells/cm^2^ of either wound area or as scaffold when size was stated.

## Data Availability

Not applicable.
